# A Rare Presentation of Adrenocortical Carcinoma

**DOI:** 10.7759/cureus.18171

**Published:** 2021-09-21

**Authors:** Mohammed Janquli, Lucy Chapman, Mary Jane Brassill

**Affiliations:** 1 Medicine, University Hospital Limerick, Limerick, IRL; 2 Ageing and Therapeutics Division, University Hospital Limerick, Limerick, IRL; 3 Medicine/Endocrinology, South Tipperary General Hospital, Clonmel, IRL

**Keywords:** management of adrenocortical carcinoma, adrenal incidentaloma investigations, cushing’s syndrome, adrenocortical carcinoma, adrenal incidentaloma

## Abstract

An adrenal incidentaloma is a mass found incidentally on radiological imaging performed for other reasons. The prevalence of these incidentalomas increases with age, and they all must be evaluated to determine if they are benign or malignant and if they are functioning or non-functioning.

A 71-year-old female presented with sub-acute bilateral lower limb pitting edema and dyspnoea. Imaging showed an 8 cm smoothly defined heterogeneous right adrenal mass and a number of low attenuation lesions throughout the liver. This case report describes a rare presentation of adrenocortical carcinoma due to an adrenal incidentaloma identified on imaging in a patient presenting with bilateral lower limb edema. The laboratory and imaging evaluation of these incidentalomas are also discussed.

## Introduction

An adrenal incidentaloma is defined as a mass greater than 1 cm found incidentally on radiological imaging performed for other reasons [1]. The term "incidentaloma" was coined by Geelhoed and Druy in 1982 who recognized that due to technological advancements in radiology, clinicians were faced with an unexpected number of early diagnosed asymptomatic adrenal lesions [2]. The prevalence of adrenal incidentalomas on imaging approaches 4.4%, while it can reach 15% in autopsies [3,4]. In a survey of 1004 cases of incidentalomas done in Italy by Mantero et al., over 70% of these incidentalomas were detected by ultrasonography (US), 28% by computed tomography (CT), and less commonly by magnetic resonance imaging (MRI). This is mainly due to the widespread use of ultrasonography to investigate abdominal pain before confirming with a higher modality imaging such as CT or MRI [5]. The prevalence varies with age ranging from <1% for patients younger than 30 years old to 7% for patients 70 years or older [1]. All adrenal incidentalomas must be evaluated to determine if they are benign or malignant and if they are functioning or non-functioning.

In this report, we present a case of adrenal incidentaloma in a patient presenting with bilateral lower limb edema. We also discuss the laboratory and radiological investigations of these incidentalomas and their management options.

## Case presentation

A 71-year-old Irish Caucasian female presented with sub-acute bilateral lower limb pitting edema and dyspnea. Her background history included obesity (BMI 45.5), atrial fibrillation, obstructive sleep apnea, subclinical hyperthyroidism, eosinophilic colitis, and diverticular disease. She was an ex-smoker with a 20 pack-year history. Furthermore, she was under surveillance for a 3 cm ovarian cyst with the local gynecology services.

**Figure 1:**
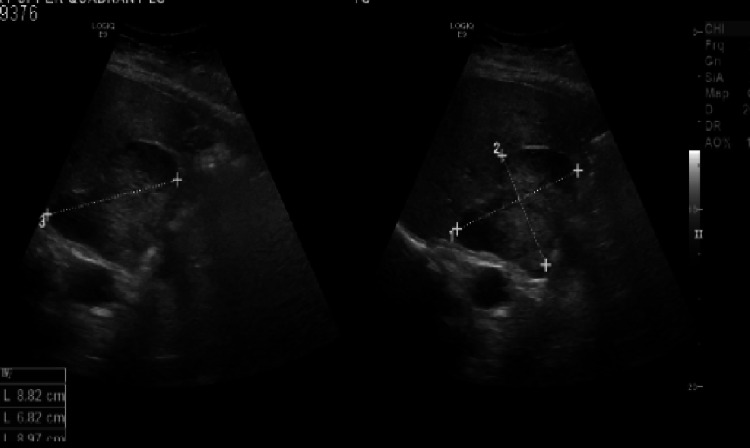
Ultrasonography showing a right adrenal mass

**Figure 2:**
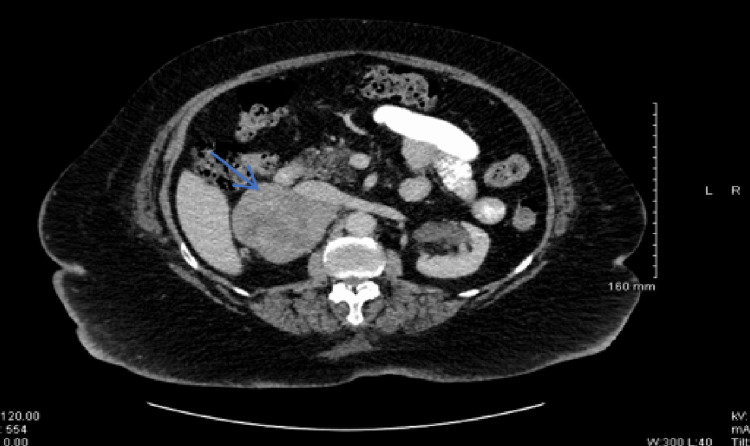
CT scan confirming the right adrenal mass (blue arrow)

The patient had normal cardiac and respiratory examinations. Her transthoracic echocardiogram showed an ejection fraction of more than 50% with normal left and right ventricles and valves. Her chest radiograph was also unremarkable. An abdominal ultrasound was performed to assess for a pelvic mass that could impede pelvic venous return leading to peripheral edema. Ultrasound revealed an 8 cm hyperechoic mass lesion at the superior pole of the right kidney as well as a 2 cm hypoechoic liver lesion (Figure 1). Subsequent computed tomography (CT) the of abdomen and pelvis with contrast confirmed an 8 cm smoothly defined heterogeneous right adrenal mass (Hounsfield Unit 57.75) and a number of low attenuation lesions throughout the liver (Figure 2).

The patient exhibited potential features of cortisol excess including weight gain of 11 kg over two months and depression. She also had a newly diagnosed type 2 diabetes mellitus prior to admission with glycated hemoglobin (HbA1c) of 50 mmol/mol. The hormonal evaluation was consistent with hypercortisolism with failure to suppress on overnight dexamethasone suppression test and elevated 24-hour urinary free cortisol (Table 1). There was no clinical or laboratory evidence of pheochromocytoma, hyperaldosteronism, or increased sex hormone production.

**Table 1:** Laboratory evaluation

Test	Result	Normal range
Serum		
Early morning cortisol	746 nmol/l	166-507
Plasma cortisol after 1 mg dexamethasone overnight (dexamethasone suppression test)	634 nmol/l	<50
Aldosterone:renin ratio	9.87	<20
Urine		
Urinary cortisol (24 h)	1506 nmol/24h	12-486
Urine noradrenaline	256 nmol/24 h	<353
Urine adrenaline	<20 nmol/24h	<50
Urine dopamine	954 nmol/24h	<2432
Urine normetadrenaline	96 nmol/24h	<281
Urine metadrenaline	<20 nmol/24h	<159

The patient was transferred to a tertiary referral center for surgical evaluation. Magnetic resonance imaging (MRI) and repeat CT thorax, abdomen, and pelvis demonstrated a large necrotic adrenal mass consistent with a primary adrenocortical carcinoma with direct invasion into the intrahepatic inferior vena cava. Multiple lung nodules concerning pulmonary metastasis were also observed. A diagnosis of adrenocortical carcinoma with pulmonary and hepatic metastasis was made. Due to metastatic disease, she was deemed unsuitable for surgery and treated pharmacologically with mitotane and metyrapone to manage symptoms caused by excess cortisol production by the tumor. She subsequently progressed to palliative care and died three months after diagnosis.

## Discussion

This case report describes a rare presentation of adrenocortical carcinoma due to an adrenal incidentaloma identified on radiological imaging in a patient presenting with bilateral lower limb edema. Multiple factors including technological advancements in imaging, availability of multiple imaging modalities in most hospitals, and increased prevalence of chronic diseases have led to a higher number of imaging tests being performed each year, which in turn have led to a higher detection rate of incidentalomas [6-8]. The presenting complaint of severe lower limb edema in our patient was likely due to direct inferior vena cava invasion impeding venous return.

Malignancy is an uncommon cause of incidentaloma in patients not known to have cancer [9]. The majority of adrenal incidentalomas (75%) are benign, non-functioning adenomas but all must be evaluated to establish if they are hormonally active and if there is any possibility of malignancy [10]. Hormonal assessment should include laboratory assessment for glucocorticoid, mineralocorticoid, or catecholamines excess as outlined in Table 1 [11]. In our patient, Cushingoid features such as facial rounding and central obesity were difficult to appreciate due to background history of obesity (body mass index 45.5 mg/m^2^) but hormonal evaluation confirmed cortisol excess.

Assessment of the risk for malignancy is largely based on lesion size and imaging characteristics. On non-contrast CT imaging, a Hounsfield Unit (HU) value of <10 is indicative of a lipid-rich, benign adenoma. Contrast-enhanced washout CT is required if a HU value of >10 is obtained. A relative washout >40% and an absolute washout >60% suggests that an adrenal lesion is benign [10]. All masses greater than 4 cm have an increased risk of malignancy and therefore should be considered for excision unless imaging features are definitively benign. If not excised additional imaging modalities or interval imaging should be considered. Adrenal biopsy does not have a role unless in the setting of known extra-adrenal malignancy where the biopsy will alter the clinical management and hormonal excess has been excluded [10,12].

Adrenal surgery is the treatment of choice for adrenocortical carcinoma. Open surgery is the standard procedure however for tumors <6 cm without local invasion laparoscopic surgery may be performed [13]. The metastatic nature of our patient’s disease resulted in pharmacological treatment alone to manage clinical symptoms related to Cushing’s syndrome with subsequent progression to palliative care.

## Conclusions

In conclusion, adrenal incidentalomas are not uncommon and require an approach combining clinical, laboratory, and radiological evaluation. It is vital to distinguish functioning from non-functioning masses and malignant from benign ones. Bilateral lower limb edema due to impedance of venous return via external compression or intravascular metastasis of the inferior vena cava can be a presenting complaint about primary adrenocortical carcinoma and should prompt clinicians for further investigations.
